# Abscess from Foreign Body in the Dog

**Published:** 1899-06

**Authors:** 


ABSCESS FROM FOREIGN BODY IN THE DOG. Patient, a pointer dog, with a suppurating fistula in the left flank. The history was that several times before at that point abscesses had formed and again cicatrized.

After a thorough exploration sufficient cause for the trouble could not be found. A counter opening was made superiorly on the side of the chest, and a seton inserted. After appropriate treatment septic infection commenced in about two weeks. There was fever, weakness, rapid emaciation, and death followed before the tract had cicatrized.

A post-mortem examination revealed that parallel with the posterior border of the last rib and embedded in the abdominal muscles was a narrow, elongated cavity, apparently closed, which was filled with a small quantity of pus and contained a piece of a stem of salt-grass, or something of a similar nature, about four inches in length. A sound could, during life, not detect this tract, nor was there any evidence of it on the exterior. How this foreign body reached this situation is not explained.

